# Special Issue: “Synthesis of Advanced Polymer Materials 2.0”

**DOI:** 10.3390/ijms252312636

**Published:** 2024-11-25

**Authors:** Haiyang Gao

**Affiliations:** PCFM Lab, GD HPPC Lab, School of Materials Science and Engineering, Sun Yat-sen University, Guangzhou 510275, China; gaohy@mail.sysu.edu.cn

## 1. Introduction

With the rapid evolution of polymer science, the field of advanced polymer materials is experiencing remarkable growth, driven by the increasing demand for versatile, high-performance materials [1]. From electronics to biomedicine and environmental applications, polymer materials are fundamental to modern innovations [2,3]. This Special Issue, “Synthesis of Advanced Polymer Materials 2.0”, presents a diverse collection of research and reviews that examine the synthesis, modification, and functionalization of polymer materials.

Researchers in the field of polymer synthesis continue to explore innovative approaches aimed at enhancing material performance, functionality, and environmental compatibility [4,5]. A persistent challenge lies in the development of polymers that effectively balance mechanical properties with functional adaptability, thereby meeting the demands of applications that range from industrial manufacturing to biomedical and electronic devices [6]. Although traditional polymers are versatile, they often fall short in specific areas, such as their mechanical properties, thermal stability, and biodegradability [7]. This issue is further complicated by the increasing demand for eco-friendly materials, as the synthesis of biodegradable or recyclable polymers presents unique chemical and engineering challenges [8]. To address critical gaps in areas such as polymer stability, processing, and environmental adaptability, this Special Issue presents new insights and methodologies that could inform future advancements in polymer science.

## 2. Overview of the Contributions to This Special Issue

The Special Issue “Synthesis of Advanced Polymer Materials 2.0” presents a diverse collection of studies that exemplify research at the forefront of polymer science. Several promising strategies for the synthesis, modification, and functionalization of several polymers are presented.

Chen and coworkers (contribution 1) present a robust epoxy resin (EP) modified with a biomass-based intumescent flame retardant. The innovative incorporation of a rigid–flexible siloxane bridge structure successfully enhances both the flame-retardant and mechanical properties of the EP. This dual-purpose approach, which combines structural toughness with dielectric stability, addresses a crucial need in high-performance polymer applications by offering safer materials without compromising flexibility.

Hernández-Fernández and Edgar Marquez (contribution 2) investigate the detrimental effects of furan residues on Ziegler–Natta catalysts during polypropylene synthesis. Through experimental and density functional theory (DFT) analyses, the study reveals how furan molecules interact with active titanium sites, thereby reducing catalytic efficiency and compromising polymer quality. This work underscores the necessity for precise control over feedstock purity and provides a theoretical foundation for future catalyst designs that exhibit greater resistance to contamination.

Miękoś and coworkers (contribution 3) introduce a novel approach to improving the properties of silicone rubber composites by applying a constant magnetic field. By aligning additives such as birch bark and expanded graphite within the silicone matrix, the team demonstrates significant enhancements in its water resistance, frost resistance, and mechanical strength. This study not only highlights the potential of magnetic fields in material synthesis, but also paves the way for the development of durable composites suitable for extreme environmental conditions.

Fabrice and coworkers (contribution 4) explore a controlled release mechanism for cinnamic acid (CA) encapsulated in Zn-Al layered double hydroxide (LDH). Utilizing π–π interactions with cinnamaldehyde (CAD), the study demonstrates a method for stabilizing CA in the interlayer space of LDH, allowing for a finely tuned release rate. This research provides an innovative framework for drug and cosmetic delivery systems, where controlled release is crucial for both efficacy and safety.

Chen and Yassar (contribution 5) examine the effects of varying side chain lengths in conjugated polymers derived from 3,4-ethylenedioxythiophene (EDOT) and diketopyrrolopyrrole (DPP) monomers. Their findings indicate that, although side chains exert minimal influence on the thermal stability of the polymers, they have a significant impact on film morphology and charge mobility, which, in turn, affects the performance of organic transistors. This study is crucial for informing the design of high-efficiency organic semiconductors.

Ghasem (contribution 6) employs a population balance model to investigate the transient behavior of a fluidized bed reactor utilized in polyethylene production. The model addresses challenges such as temperature fluctuations and reactor instability. The implementation of Proportional Integral Derivative (PID) control strategies enhances system stability, ensuring more consistent polymer properties. This work underscores the significance of dynamic control mechanisms in large-scale polymerization processes, particularly for materials like polyethylene, where process stability is essential.

Gao and coworkers (contribution 7) provide a comprehensive review of recent advancements in the synthesis of non-alternating polyketones through CO and ethylene copolymerization. This review summarizes novel catalyst systems, including phosphine–sulfonate and phosphinophenolate complexes, which enable precise control over the chain structure and degradation behavior. This review serves as a valuable resource for researchers aiming to develop high-performance, environmentally friendly polyketone materials.

## 3. Conclusions

The articles in this Special Issue highlight the necessity for innovative approaches in the field of polymer materials. As we look to the future, there are several promising research directions that are fundamental in this field:

(1) Synthesis of new polymers: The development of new polymers is a persistent issue that must be addressed in order to meet the growing demand for versatile and high-performance polymer materials, especially the synthesis of new functional polymers and the functionalization of old polymers [9].
(2) Control over molecular architecture: The properties of polymers are significantly influenced by their molecular architecture. The development of new synthetic methods and technologies enables more precise control over polymer structures [5,10].
(3) Sustainable and biodegradable polymers: The synthesis of polymers with a reduced environmental impact, including biodegradable and recyclable options, remains a high priority. Research on polymer composites with natural additives or polymers derived from renewable sources is essential for sustainable development [11].
